# CRISPR/Cas9 mediated targeted mutagenesis of the fast growing cyanobacterium *Synechococcus elongatus* UTEX 2973

**DOI:** 10.1186/s12934-016-0514-7

**Published:** 2016-06-23

**Authors:** Kristen E. Wendt, Justin Ungerer, Ryan E. Cobb, Huimin Zhao, Himadri B. Pakrasi

**Affiliations:** Department of Biology, Washington University, St. Louis, MO 63130 USA; Department of Chemical and Biomolecular Engineering, University of Illinois at Urbana-Champaign, Urbana, IL 61801 USA

**Keywords:** Cyanobacteria, *Synechococcus*, CRISPR, Cas9, Genome modification

## Abstract

**Background:**

As autotrophic prokaryotes, cyanobacteria are ideal chassis organisms for sustainable production of various useful compounds. The newly characterized cyanobacterium *Synechococcus elongatus* UTEX 2973 is a promising candidate for serving as a microbial cell factory because of its unusually rapid growth rate. Here, we seek to develop a genetic toolkit that enables extensive genomic engineering of *Synechococcus* 2973 by implementing a CRISPR/Cas9 editing system. We targeted the *nblA* gene because of its important role in biological response to nitrogen deprivation conditions.

**Results:**

First, we determined that the *Streptococcus pyogenes* Cas9 enzyme is toxic in cyanobacteria, and conjugational transfer of stable, replicating constructs containing the *cas9* gene resulted in lethality. However, after switching to a vector that permitted transient expression of the *cas9* gene, we achieved markerless editing in 100 % of cyanobacterial exconjugants after the first patch. Moreover, we could readily cure the organisms of antibiotic resistance, resulting in a markerless deletion strain.

**Conclusions:**

High expression levels of the Cas9 protein in *Synechococcus* 2973 appear to be toxic and result in cell death. However, introduction of a CRISPR/Cas9 genome editing system on a plasmid backbone that leads to transient *cas9* expression allowed for efficient markerless genome editing in a wild type genetic background.

**Electronic supplementary material:**

The online version of this article (doi:10.1186/s12934-016-0514-7) contains supplementary material, which is available to authorized users.

## Background

Photosynthetic microbes are of considerable interest for applications in carbon sequestration, photosynthetic production of fuels, and biosynthesis of other valuable chemicals such as pharmaceuticals [1, 2]. The advantage of using cyanobacteria as biofactories is that they grow on CO_2_ and sunlight alone; this reduces greenhouse gas emissions and decreases dependence on petroleum-based products. Furthermore, cyanobacteria are the evolutionary ancestors of plastids and serve as model organisms for the study of the photosynthetic apparatus. Commonly studied cyanobacteria such as *Synechococcus elongatus* PCC 7942, *Synechococcus* sp. PCC 7002, and *Synechocystis* sp. PCC 6803 have been genetically engineered to generate a variety of useful products including ethylene [3], hydrogen [4], free fatty acids [5], ethanol [6], and isoprene [7]. Additionally, genetic manipulation has been used to rewire central metabolism and redirect carbon sequestration into end products by deleting competing pathways [8, 9].

A newly identified cyanobacterial strain that has the potential to become a versatile chassis for metabolic engineering and biological discovery is *Synechococcus* 2973. With a 1.9-h doubling time, *Synechococcus* 2973 has a growth rate akin to that of *Saccharomyces cerevisiae* [10]. The genome sequence of *Synechococcus* 2973 is 99.8 % identical to that of the model organism *Synechococcus* 7942, which has a slower doubling time of 4.9 h. However, the development of *Synechococcus* 2973 as a model organism has been hindered by the lack of an efficient genetic modification system. *Synechococcus* 7942 is naturally competent, whereas *Synechococcus* 2973 does not have the capacity to take up naked DNA. Although *Synechococcus* 2973 has proven to be capable of conjugative transfer of DNA, the rate at which subsequent genome modification occurs is less than that found in *Synechococcus* 7942 and other model species of cyanobacteria.

The current genetic manipulation system for *Synechococcus* species is well developed, however, it often requires a significant amount of time to generate the desired mutant strains. The strategy typically used for engineering a deletion mutant in *Synechococcus* relies on double homologous recombination between a suicide vector and host chromosome, and involves replacing the gene of interest with a selective marker [11]. Additional genetic alterations are made by integrating other antibiotic resistance markers. This restricts pathway engineering because there are a limited number of antibiotic cassettes available. Moreover, cyanobacteria maintain multiple copies of their chromosome and numerous rounds of segregation are often necessary to obtain a completely segregated mutant [12]. Although the genome copy number for *Synechococcus* 2973 is yet to be determined, *Synechococcus* 7942 cells contain three to four genome copies. As a result, the process of segregation can take weeks of restreaking on selective media to obtain a segregated strain.

Alternatively, markerless deletion strategies have been developed that rely on a dominant streptomycin-sensitive *rps12* mutation [13]. A major drawback of this system is that it requires working in a genetic background that contains the appropriate *rps12* mutation. Additionally, this strategy is time-consuming because it relies on two subsequent rounds of transformation. Recently, CRISPR/Cas9 systems have emerged as versatile editing platforms for engineering markerless mutations in a wide variety of organisms [14–16]. However, a CRISPR/Cas9 system for markerless genome editing in cyanobacteria has not yet been developed.

In nature, the CRISPR (clustered regularly interspaced short palindromic repeats)/Cas9 (CRISPR associated protein 9) system provides adaptive immunity in bacteria against invading viruses or plasmids by cleaving and degrading the exogenous DNA [17]. Upon infection, invader sequences are incorporated as spacers between a series of palindromic repeats in a CRISPR array [18, 19]. The CRISPR array transcripts are then processed into two RNA components: the crRNA and tracrRNA [20]. These are used to guide the Cas9 nuclease to the complementary target sequence, where Cas9 creates a double stranded break [18]. The CRISPR system can be engineered for genome editing by reprogramming the spacer sequences to be complementary to the genetic target [21]. The directed break is subsequently repaired by double homologous recombination, during which a homologous sequence serves as a repair template [22, 23]. By providing a repair template containing the desired change to the target sequence, specific genomic mutations or deletions may be made at the cut site. Although there are not yet any instances of application of a CRISPR/Cas9 genome editing system in cyanobacteria, studies have been performed that aim to characterize the native cyanobacterial CRISPR system [24, 25]. Little work has been performed to characterize the CRISPR/Cas system in *Synechococcus elongatus*. However, computational analysis of various cyanobacterial genomes has predicted the presence of various combinations of IA, IB, IIA, IIB, IIC, IID, IIE, IIF, IIG, and III CRISPR/Cas subtypes [24, 26]. Furthermore, recent studies have been aimed at developing a CRISPRi system for gene repression in cyanobacteria [27].

In the current study, we repurposed a CRISPR/Cas9 system, originally developed for genome editing in *Streptomyceslividans*, for use in the fast-growing cyanobacterium *Synechococcus* 2973 [28]. Using derivatives of the pCRISPomyces plasmids, we introduced a markerless deletion into *Synechococcus* 2973 and determined that the mutated strain was completely segregated in the first patch. In addition to serving as metabolic chassis, cyanobacteria are ideal systems for better understanding photosynthetic processes [29]. Thus, as a proof of concept for the ability to generate a markerless deletion mutant with the CRISPR/Cas9 system, we chose to target the *nblA* gene, which has an important function in cellular response to nutrient deprivation conditions. Cyanobacteria have large antenna protein complexes that harvest light for photosynthesis [30]. One intriguing feature of these prokaryotes is their ability to modulate the size and structure of these antenna complexes based on nutrient availability [31]. NblA is involved in the degradation of phycobilisomes, one of the primary antenna protein complexes associated with photosystem II [32]. By targeting *nblA* for deletion, we demonstrate that the CRISPR/Cas9 system can be used to better characterize the function of biologically important genes.

This improved method of genome editing is expected to facilitate rapid and efficient genetic engineering of *Synechococcus* strains. Moreover, the number of edits that can be made using CRISPR/Cas9 editing is not limited by choice of antibiotic cassettes and will enable extensive modification of host genomes for the production of useful bioproducts.

## Results

### Developing an RSF1010-based CRISPR/Cas9 system

We initially attempted to assemble a complete CRISPR system using the medium copy number plasmid backbone pVZ321, an RSF1010 based backbone, which is stably maintained in cyanobacteria [33, 34]. We selected the *nblA* gene, an essential element for phycobilisome degradation in *Synechococcus* 2973, as a target for deletion [32]. These mutants have a phenotype that can be detected visually. While the wild type *Synechococcus* 2973 strain exhibits yellow bleaching that is characteristic of phycobilisome degradation when grown in media lacking nitrate, the ∆*nblA* strain has an obvious non-bleaching phenotype and remains green under these conditions. Furthermore, bleaching is only apparent when all copies of *nblA* have been deleted, allowing it to serve a visual marker for segregation.

The construct pVZ321 was engineered to contain *S. pyogenes cas9* (derived from pCRISPomyces-2), a synthetic guide RNA (sgRNA) designed to target *nblA*, and an editing template to introduce the *nblA* deletion. After multiple conjugation attempts with this construct, we were unable to recover exconjugants. However, the same backbone lacking the CRISPR/Cas9 system yielded ~250 colonies in each of two conjugation attempts. To assay for *cas9* toxicity, we proceeded by engineering the pVZ321 backbone to contain only *cas9*. Once again we were unable to recover colonies from conjugation with the *cas9* containing plasmid. In an effort to circumnavigate toxicity from *cas9*, we reduced expression to basal level by removing 500 base pairs of the upstream sequence, including the ribosome binding site and promoter. Conjugation of the resulting plasmid into *Synechococcus* 2973 yielded less than five exconjugants in each of two attempts, while the vector without *cas9* yielded ~250 exconjugants.

### Applying the pCRISPomyces-2 CRISPR/Cas9 system

**Fig. 1:**
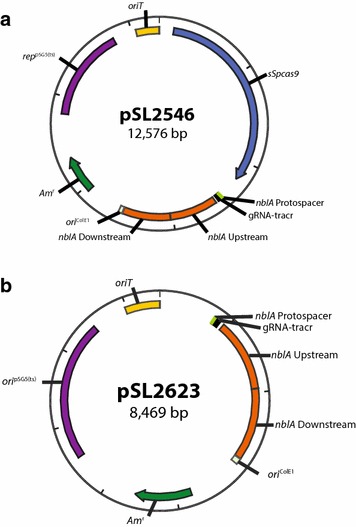
Plasmids were generated using pCRISPomyces-2 backbone to engineer the ∆*nblA* line. **a** The *nblA* deletion plasmid including *cas9* and **b** the *nblA* editing plasmid excluding *cas9* are depicted

Experiencing little success with the pVZ321 backbone, we switched to a vector that would theoretically allow for transient expression of *cas9*: the pCRISPomyces-2 construct from the Zhao lab. Replication of this vector is dependent on a *Streptomyces ghanaensis* pSG5 origin of replication, which is not functional at temperatures above 34 °C [35]. In our study, all conjugation experiments were performed 38 °C (the optimal temperature for *Synechococcus* 2973), which is above the permissive replication temperature in *S.ghanaensis*. This allows for initial transient expression of *cas9* directly after its conjugation into *Synechococcus* 2973, but prevents prolonged expression of the toxic gene because the plasmid presumably does not replicate after it is transformed into the cyanobacteria. We modified the pCRISPomyces-2 vector to target *nblA* in *Synechococcus* 2973 by inserting an sgRNA that targets *nblA* and an editing template designed to introduce the *nblA* deletion (Fig. 1a).

**Fig. 2:**
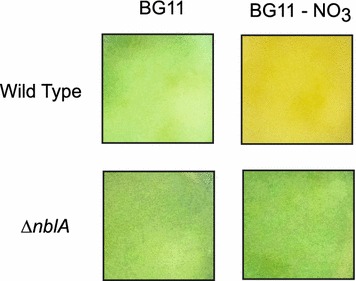
*Synechococcus* 2973 exconjugants do not exhibit characteristic bleaching under nitrogen deprivation conditions. Triparental mating was used to introduce the ∆*nblA* CRISPR/Cas9 plasmid into *Synechococcus* 2973. Exconjugants were patched onto selective media and then transferred to liquid cultures in standard and nitrogen deprivation conditions

We employed a deletion strategy that would not rely on the integration of a selective marker into the genome as a proof-of-concept for making a markerless genome modification. Triparental mating was used to introduce the ∆*nblA* CRISPR construct into *Synechococcus* 2973. Antibiotic selection was used to force temporary persistence of the plasmid at a basal level. A typical conjugation yielded 21 colonies and a subset of these exconjugants was assayed for bleaching in media lacking nitrate (Fig. 2). Additionally, PCR and Sanger sequencing were used to confirm that the non-bleaching phenotype was the result of *nblA* deletion and not a single recombination event in colonies that failed to bleach under nitrogen deprivation conditions.

### Evaluating CRISPR/Cas9 mediated editing

**Fig. 3:**
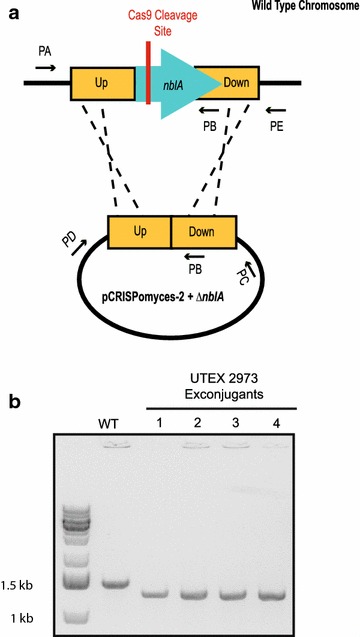
Double homologous recombination was used to generate the markerless deletion. **a** Schematic of the double homologous recombination event that results in deletion of *nblA* from the chromosome is shown. *Black arrows* indicate primers, *yellow* *rectangles* indicate homology arms, and the *blue* *arrow* represents the *nblA* gene. **b** PCR was used to confirm the deletion of *nblA*. Colony PCR using Primer A and Primer B of mutant *Synechococcus* 2973 yielded a product that is 180 base pairs lower in molecular weight than the band produced by wild type culture

To determine the proportion of editing that is dependent on Cas9 cleavage, we built a secondary construct in which the *cas9* gene was deleted (Fig. 1b). Triparental mating was used to introduce the -*cas9* construct into *Synechococcus* 2973 to compare differential exconjugant yields. We used PCR to assess whether exconjugants were edited and segregated (Table 1; Fig. 3).

**Table 1:** Conjugation results showing that the plasmid backbone has an effect on achieving successful editing

Construct	Total number of exconjugants	Percent of exconjugants edited and segregated on 1st patch
pVZ321	>250	–
pVZ321 + *sSpcas9*	0	–
pVZ321 + *nblA* sgRNA, ∆*nblA* editing template, *sSpcas9*	0	N/A
pVZ321 + *nblA* sgRNA, ∆*nblA* editing template, *sSpcas9* (no promoter)	0	N/A
pVZ321 + *nblA* sgRNA, ∆*nblA* editing template, *sSpcas9* (no RBS)	4	0/4 (0 %)
pCRISPomyces-2 + *nblA* sgRNA, ∆*nblA* editing template, *sSpcas9*	21	16/16 (100 %)
pCRISPomyces-2 + *nblA* sgRNA, ∆*nblA* repair template—*sSpcas9*	32	3/10 (30 %)

**Fig. 4:**
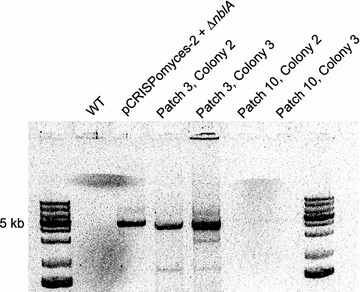
The *cas9* gene was present in early patches, but absent once curing had occurred. Primers were designed to sit on the pCRISPomyces-2 backbone, outside *cas9*, yielding a 4.252 kb product if the plasmid was present

The presence of antibiotic resistant exconjugants suggested that the editing plasmid was being maintained, even under conditions that were supposedly non-permissive for replication. We confirmed this by performing PCR assays to test for the presence of *cas9* in exconjugants (Fig. 4).

### Assessing the potential to cure edited strains of CRISPR plasmid machinery

**Fig. 5:**
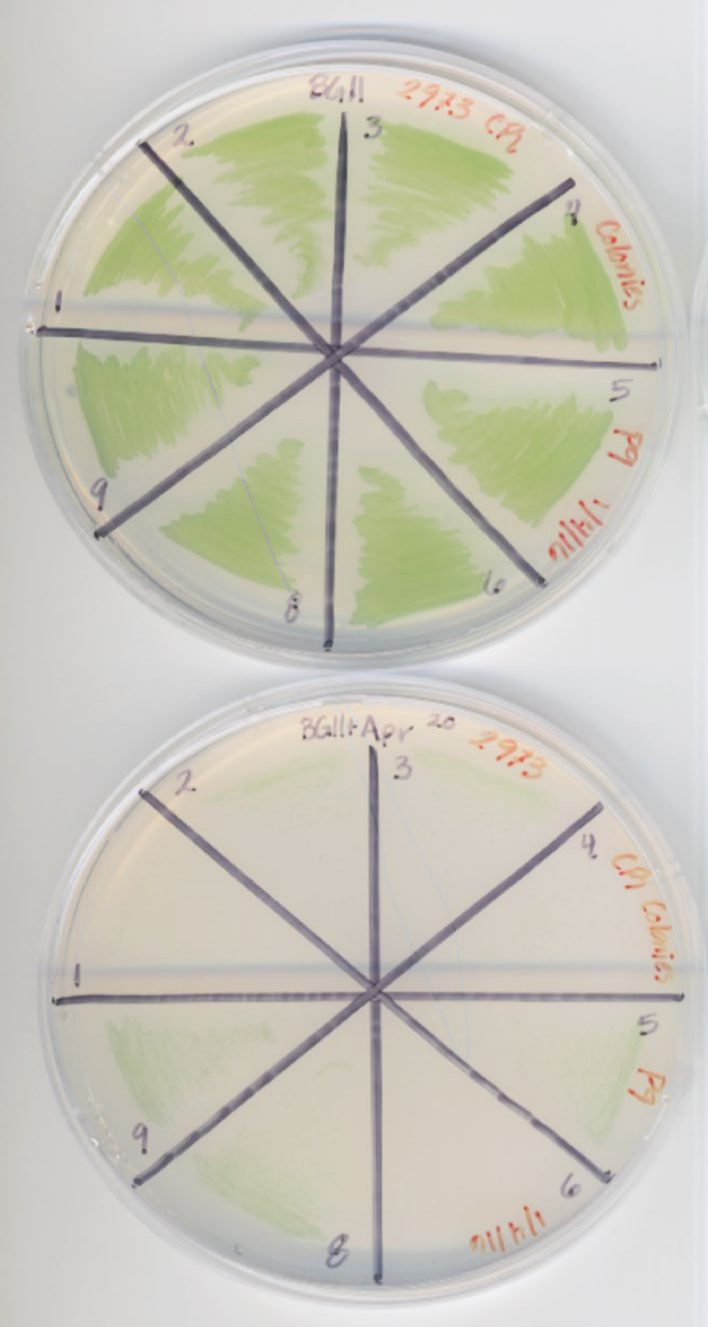
Curing of the CRISPR ∆*nblA* editing plasmid during consecutive serial patching. *Top plate* exhibits the growth of nine exconjugants on nonselective medium, and *bottom plate* shows the absence of growth on media containing apramycin. By patch ten, no growth is seen on the antibiotic-containing plate

In order to determine whether exconjugants could be cured of *cas9* and the apramycin-resistance marker, colonies were patched onto media lacking antibiotic selection. The loss of the ability to grow on antibiotic-containing media occurred by patch ten (Fig. 5). Furthermore, after the organisms lost their capacity to grow on selective medium, we used PCR to assay for the presence of *cas9*, and found that we were unable to amplify the gene in patch ten (Fig. 4).

## Discussion

### Cas9 is toxic in *Synechococcus* 2973

The inability to produce a significant number of exconjugants with constructs containing *cas9* suggests that the gene is toxic in *Synechococcus* 2973 when it is introduced on a medium copy number plasmid. The fact that only five colonies were yielded from conjugation with a construct in which the *cas9* RBS was removed (compared to the ~250 colonies with the construct lacking *cas9*) suggests that these exconjugants are “escapees” with respect to *cas9* toxicity. Furthermore, we conclude that the *cas9* gene cannot be stably maintained in *Synechococcus* at a medium copy number level. Although the reason behind Cas9 toxicity is currently unclear, one possibility is that *S. pyogenes* Cas9 has off-target effects in cyanobacterial cells. The enzyme may be cleaving genomic DNA in regions other than those targeted by the synthetic sgRNA, and that the cell is unable to repair these breaks, thus resulting in lethality.

### Transient *cas9* expression achieves genome editing

After switching to a plasmid backbone that facilitated transient expression of *cas9* (pCRISPomyces-2), we found that we were able to engineer the intended *nblA* deletion strain. All of the exconjugants failed to bleach under nitrogen deprivation conditions, suggesting that *nblA* had been edited. Since the organism cannot retain any functional copies of *nblA* to exhibit the non-bleaching phenotype, this also suggests that segregation had occurred and only the mutated genome copy remained.

The fact that we were able to produce antibiotic-resistant exconjugants shows that permissive temperature of replication differs between the *Synechococcus* 2973 system and the *Streptomyces lividans* system. We believe that the pCRISPomyces-2 backbone replicates at a basal level at 38 °C in *Synechococcus* 2973. PCR assays show that *cas9* was present in early patches, but not in later patches (Fig. 4), making it transient in nature.

### The presence of *cas9* improves genome editing efficiency

Although the total number of exconjugants yielded with a construct lacking *cas9* was higher, which may be attributed to the smaller size of the construct, the rate of editing and segregation in the absence of *cas9* was reduced. When using a construct that contains *cas9*, exconjugants are edited and segregated 100 % of the time in the first patch. However, constructs lacking *cas9* are only edited and segregated 30 % of the time. This suggests that Cas9-mediated cleavage accounted for roughly 70 % of editing in the engineered *Synechococcus* 2973 cells.

### Edited exconjugants can be cured of the CRISPR plasmid machinery

Many bacterial CRISPR/Cas9 systems rely on the generation of a strain that has *S. pyogenes cas9* engineered into the genome of the organism undergoing editing. However, with this system, edits are made in a genetic background that is distinct from the wild type organism. The benefit of introducing the CRISPR/Cas9 editing machinery on a plasmid is that after editing is completed, the foreign construct may be cured from the organism, leaving behind a truly “markerless” modification in a wild type background. Furthermore, the fact that *cas9* appears to be toxic in cyanobacteria suggests that engineering *cas9* into the genome is a suboptimal approach for this class of organisms.

The fact that the exconjugants lost the ability to grow on selective media after subsequent rounds of patching suggests that the organisms were cured of the plasmid containing the CRISPR/Cas9 machinery. Furthermore, the inability to amplify the antibiotic resistance gene after passaging on nonselective medium provides further confirmation that the organisms were cured of the plasmid. Thus, we were able to take advantage of one of the most valuable aspects of CRISPR/Cas9 genome editing: ability to generate markerless genetic modifications in a wild type background.

## Conclusions

This is the first report of the use of a CRISPR/Cas9 genome editing system in a cyanobacterial strain. Although *cas9* has been used with great success to make genome modifications in other organisms, we found that in *Synechococcus* 2973, *cas9* expression must occur in a transient manner to achieve successful editing. The fact that editing success depends on transient *cas9* expression in one cyanobacterial strain suggests that *cas9* toxicity may be the reason the application of CRISPR/Cas9 genome editing in cyanobacteria has lagged behind that of other organisms. The CRISPR/Cas9 genome editing method described here will no doubt advance diverse scientific investigation in cyanobacteria.

## Methods

### Bacterial strains and culture conditions

All cloning was performed in the *Escherichiacoli* strains HB101 and XL1-Blue. Cells were grown at 37 °C in LB media in liquid or on agar plates supplemented with 50 μg/mL apramycin or 50 μg/mL kanamycin as required. *Synechococcus* 2973 and *Synechococcus* 7942 cells were grown in BG11 medium at 38 °C under 80 μE m^−2^s^−1^ of continuous white light in two manners: on agar plates, supplemented with 20–50 μg/mL apramycin as needed or shaking in 125 mL Erlenmeyer flasks.

### Conjugation of pCRISPomyces-2 based *nblA* editing plasmid into *Synechococcus* 2973

Plasmids used for conjugation were constructed as described in the Additional file 1 addendum. Tri-parental mating was used to introduce the *nblA*-targeting pCRISPomyces-2 into wild type *Synechococcus* 2973, with pRL443 as the conjugal plasmid and pRL623 as the helper plasmid [36]. The HB101 strain, which already carried pRL623, was transformed with *nblA*-targeting pCRISPomyces-2 and served as the cargo-carrying strain in the tri-parental mating. *Escherichiacoli* cultures were inoculated approximately 17 h prior to use and grown to OD600 = 0.6. Cyanobacterial strains were also inoculated approximately 17 h prior to use to OD730 = 0.25, and grown to OD730 = 0.4, as measured on a μQuant Bio-Tek plate reader. All bacterial cultures were washed prior to use in conjugation with either distilled water for *E. coli* or BG11 for cyanobacteria. 100 μL of the cargo and conjugal *E. coli* lines were combined with cyanobacteria cells from 1 mL of liquid culture for each conjugation reaction and resuspended in a total volume of 300 μL. Subsequently, 100 μL of the conjugation reaction was plated on BG11 agar plates containing HATF transfer membranes (Millipore). In conjugation with pVZ321-based plasmids, filters were incubated on nonselective media for 24 h before transferring the membranes to BG11 agar plates supplemented with 50 μg/mL kanamycin. For conjugation with pCRISPomyces-2- based plasmids, after 4 days, the membranes were moved to BG11 agar plates containing 20 μg/mL apramycin, and after 3 more days these membranes were transferred to BG11 agar plates containing 50 μg/mL apramycin.

### Assessing bleaching under nitrogen deprivation conditions

Exconjugants were transferred from patches to liquid cultures and allowed to grow to a suitable volume and density to allow for visualization of bleaching. Cultures were washed three times with BG11 lacking nitrate, resuspended in the same media, and assessed 24 h later for differential coloration in comparison to a wild-type sample.

### PCR assays to confirm accurate editing

DreamTaq (Thermo Fisher Scientific) was used for all deletion confirmation reactions in addition to all of the reactions checking for single recombination events. The set composed of PrimerA/PrimerB was used to check for deletion of *nblA* in the chromosome, the PrimerA/PrimerC set was used to check for single recombinants in one orientation, and the PrimerD/PrimerE set was used to check for single recombinants in the other possible orientation (see Fig. 3).

### Curing the CRISPR plasmid from edited strains

Exconjugants were initially selected for on BG11 agar plates supplemented with 20 μg/mL apramycin. The first patch was carried out on BG11 agar plates supplemented with 20 μg/mL apramycin. Subsequent patches were performed on BG11 agar plates. Colonies were assayed for curing by the loss of ability to grow on apramycin-containing media after each round of patching. After colonies appeared to be cured, further testing was done via colony PCR, to ensure that a portion of the pCRISPomyces-2 backbone could not be amplified from later patches as opposed to amplification in earlier patches (Cas9ChkF/Cas9ChkR).

## Additional file

10.1186/s12934-016-0514-7 The supporting information for this document includes additional details on vector construction, the sequences for all of the primers used in this study, the annealing temperatures for all of the primer sets used for amplification in this study, and a list of the constructs used in this study with descriptions of their molecular and genetic contents.

## Abbreviations

Cas: CRISPR associated protein
CRISPR: clustered regularly interspaced short palindromic repeats
PCR: polymerase chain reaction
sgRNA: synthetic guide RNA
