# Supplementary material for: CRISPR/Cas9 mediated targeted mutagenesis of the fast growing cyanobacterium Synechococcus elongatus UTEX 2973
Source: Microb Cell Fact. 2016 Jun 23;15:115. doi: 10.1186/s12934-016-0514-7 (PMC4917971; doi:10.1186/s12934-016-0514-7)
Supplement: Supplementary file 1 — 10.1186/s12934-016-0514-7 The supporting information for this document includes additional details on vector construction, the sequences for all of the primers used in this study, the annealing temperatures for all of the primer sets used for amplification in this study, and a list of the constructs used in this study with descriptions of their molecular and genetic contents. [file 12934_2016_514_MOESM1_ESM.docx]

**Supporting Information**

**CRISPR/Cas9 mediated targeted mutagenesis of the fast growing cyanobacterium *Synechococcus elongatus* UTEX 2973**

Kristen E. Wendt^1^, Justin Ungerer^1^, Ryan E. Cobb^2^, Huimin Zhao^2^ and Himadri B. Pakrasi^1^

^1^Department of Biology, Washington University, St. Louis, MO 63130; ^2^Department of Chemical and Biomolecular Engineering, University of Illinois at Urbana-Champaign, Urbana, IL 61801

**Table of Contents**

**Plasmid Assembly Methods**……………………………………………………………………………… 2

**Table S1.** List of plasmids used in this study……………………………………………………………… 3

**Table S2.** Annealing Temperatures for PCR Reactions Performed in This Study………………………… 4

**Table S3.** List of Plasmids Used in This Study……………………………………………………………. 5

**Plasmid Assembly Methods**

**Construction of pVZ321 based vectors for *nblA* targeting.** To construct a CRISPR system that would replicate in cyanobacteria, we excised the CRISPR region containing *cas9* and the gRNA from pCRISPomyces-2 using *Apa*LI and *Avr*II. The resulting fragment was cloned into the replicating plasmid pVZ321 by cutting with *Apa*LI and *Xba*I enzymes*,* yielding pSL2545*.* The pSL2545 contained *Bbs*I sites that were converted to *Aar*I sites for cloning in required protospacers. PCR was performed to amplify the gRNA region of pSL2545 with primers aarI1/aarI3, aarI2/aarI4, and aarI5/pJU18R, which contain the necessary sequence modifications to introduce the *Aar*I sites. The three fragments containing the upstream region, *lacZ*, and downstream region were assembled using overlapping extension PCR, and the resulting DNA was used to replace the same gRNA section of pSL2545 using *Eco*RI and *Xba*I enzymes to yield pSL2610. Protospacers (20 nucleotides) were designed to target *nblA,* were generated by phosphorylating and ligating the oligonucleotides nblAgRNAL and nblAgRNAR. The resulting DNA contained overhangs matching those that result after excising *lacZ* from pSL2610 with *Aar*II. Colonies that contained the protospacer were identified by plating on LB supplemented with X-Gal and IPTG for blue/white screening and were further verified by sequencing. The ∆*nblA* construct was named pSL2546. Repair regions containing the *nblA* deletion were generated with PCR. The *nblA* deletion repair region was amplified out of pSL2470 using the nblAupstreamF/nblAdownstreamR primers and was cut using *Spe*I and cloned into the *Xba*I site on pSL2545, creating final plasmid to introduce the *nblA* deletion (pSL2566).

Plasmids containing only *cas9*, promoterless *cas9*, or *cas9* lacking a RBS were made by amplifying *cas9* out of pCRISPomyces-2 using the primer pairs cas9L/cas9R, cas9-PapaLI/cas9R, or cas9-RBS-L/cas9R. The resulting fragments were cloned into pVZ321 using *Apa*LI/*Eco*RI to yield pSL2672, pSL2677, and pSL2658. As a control pVZ321 was cut with *Apa*LI/*Eco*RI, blunted, and recircularized.

**Construction of pCRISPomyces-2 based vectors for *nblA* targeting.** The *∆nblA* construct is a derivative of pCRISPomyces-2 (24). The *nblA* editing template was generated by amplifying the 1000 base pairs directly upstream and downstream of genomic *nblA* in *Synechococcus* 2973 with NblA_Upstream_F/NblA_Upstream_R primers and NblA_Downstream_F/NblA_Downstream_R primers with Phusion polymerase. NblA_Upstream_R and NblA_Downstream_F were designed with overlapping ten basepair tails coding for the *Bam*HI restriction enzyme recognition site, allowing the upstream and downstream homology arms to be ligated to one another and thus generating the deletion repair template. PCR with Phusion polymerase and the NblA_Upstream_F/NblA_Downstream_R primer set was used to amplify the deletion fragment from the ligation reaction mixture, which was then gel purified. NblA_Upstream_F and NblA_Downstream_R were designed with tails specific to *Bcu*I (9 basepairs) for cloning into pCRISPomyces-2. The editing template was ligated into the *Bcu*I restriction site in pCRISPomyces-2 and HB101 *E. coli* were transformed with the ligation mixture. Sanger sequencing was used to confirm the presence and orientation of the ∆*nblA* fragment.

The *nblA*-targeting sgRNA was introduced into the plasmid via Golden Gate Assembly with the nblA_proto_F and nblA_proto_R as described in Cobb, Wang, and Zhao (2016) *^1^*. The pCRISPomyces-2 construct containing the *nblA* editing template and sgRNA became pSL2546.

The *nblA* editing plasmid that lacks *cas9* was generated by using PCR with the cas9less_F/cas9less_R primer and KlenTaq polymerase set to amplify the pSL2546 backbone excluding *cas9*. This PCR product was then gel purified and treated with T4 polynucleotide kinase followed by ligase to promote self-circularization and create pSL2623. The ligation mixture was then used to transform chemically competent XL1-Blue cells. Transformants were selected on apramycin-containing media. Plasmid from transformed colonies sequenced to ensure that the intended construct was generated.

*^1^* Cobb, R. E.; Wang, Y.; Zhao, H., High-efficiency multiplex genome editing of *Streptomyces* species using an engineered CRISPR/Cas system. *ACS Synthetic Biology* **2014,** *4* (6), 723-728.

| **Table S1.** List of primers used in this study | |
| --- | --- |
| **Name** | **Sequence** |
| aarI1 | ATTAGAATTCAGATCTACGCGTTC |
| aarI2 | GTATCTGAAAGGGGATACGCTCATGCAGGTGTCAGCCGCTACAGGGCGCGT |
| aarI3 | ACGCGCCCTGTAGCGGCTGACACCTGCATGAGCGTATCCCCTTTCAGATAC |
| aarI4 | CTTGCTATTTCTAGCTCTAAAACTCATGCAGGTGACCGGTGGAAAGCGGGCA |
| aarI5 | TGCCCGCTTTCCACCGGTCACCTGCATGAGTTTTAGAGCTAGAAATAGCAAG |
| pJU18R | AATAGGCGTATCACGAGG |
| nblAgRNAL | ACGCTCAGCAGGTGCGTGACATCT |
| nblAgRNAR | AAACAGATGTCACGCACCTGCTGA |
| cas9L | AATAGAATTCGGCGTCGTGGACTATGAG |
| cas9R | CGGTCGCTGAGGCTTGCAG |
| cas9-PapaLI | GAATGTGCACCGAGTAGACGACGGAGACG |
| Cas9-RBS-L | GAATGTGCACATGGACAAGAAGTACAGCATCG |
| NblA_Upstream _F | GGACTAGTTGCTGATCCTGCTGCCCAT |
| NblA_Upstream_R | CGGGATCCTGGGAGCCTCCGGCAC |
| NblA_Downstream_F | CGGGATCCACCGTGTGCAAGACTTGCCC |
| NblA_Downstream_R | CTACTAGTCCATCCCCATCCAGTCGATCGC |
| nblA_proto_F | AAACTCAGCAGGTGCGTGACATCT |
| nblA_proto_R | ACGCAGATGTCACGCACCTGCTGA |
| cas9less_F | GCCGATGCTGTACTTCTTGTCCA |
| cas9less_R | CAAAAGCGGCCTTTGACTCCC |
| Primer A | GCTGGCCCCAGTCCATCG |
| Primer B | TGCCCGGAAGTTCAACAGCTC |
| Primer C | ACGATAGTTACCGGATAAGGCGC |
| Primer D | TTATACGGCTGCCAGATAAGGCTTG |
| Primer E | CCTGTACAAAGGCCGCAATCGA |
| Cas9ChkF | GCGCGCACAGCCTTGAA |
| Cas9ChkR | CCGATATATTCGGTCGCTGAGG |

| **Table S2.** Annealing Temperatures for PCR Reactions Performed in This Study | |
| --- | --- |
| **Primer Set** | **Annealing Temperature** |
| PrimerA/PrimerB | 57.4°C |
| PrimerA/PrimerC | 55.8°C |
| PrimerD/PrimerE | 55.7°C |
| AprChkF/AprChkR | 55.6°C |
| Cas9ChkF/Cas9ChkR | 54.2°C |
| aarI1/aarI3 | 60.0°C |
| aarI2/aarI4 | 60.0°C |
| aarI5/pJU18R | 60.0°C |
| nblAupstreamF/nblAdownstreamR | 56.0°C |
| cas9L/cas9R | 56.0°C |
| cas9-PapaLI/cas9R | 56.0°C |
| cas9-RBS-L/cas9R | 56.0°C |
| NblA_Upstream_F/NblA_Upstream_R | 69.0°C |
| NblA_Downstream_F/NblA_Downstream_R | 72.0°C |
| NblA_Upstream_F/NblA_Downstream_R | 72.0°C |
| cas9less_F/cas9less_R | 62.0°C |

| **Table S3.** List of Plasmids Used in This Study | | |
| --- | --- | --- |
| **Plasmid** | **Description** | **Source or Reference** |
| pVZ321 | RSF1010 *repA, repB, repC, mobA, mobC*, RSF1010 *oriV*, *Km*^r^ | *^a^* |
| pSL2545 | pVZ321 + *nblA* sgRNA, *sSpcas9* | this study |
| pSL2610 | pVZ321 + *sSpcas9*, *lacZ* | this study |
| pSL2546 | pVZ321 + *nblA* sgRNA, *sSpcas9* | this study |
| pSL2566 | pVZ321 + ∆*nblA* editing template, *nblA* sgRNA, *sSpcas9* | this study |
| pSL2672 | pVZ321 + *sSpcas9* (no promoter) | this study |
| pSL2677 | pVZ321 + *sSpcas9* (no RBS) | this study |
| pCRISPomyces-2 | *Am*r, *oriT*, *rep*pSG5(ts), *ori*ColE1, *sSpcas9*, sgRNA cassette | Zhao Lab*^b^* |
| pSL2546 | pCRISPomyces-2 + ∆*nblA* editing template, *nblA* sgRNA | this study |
| pSL2623 | pCRISPomyces-2 + ∆*nblA* editing template, *nblA* sgRNA – *sSpcas9* | this study |

*^a^*Zinchenko, V.; Piven, I.; Melnik, V.; Shestakov, S., Vectors for the complementation analysis of cyanobacterial mutants. *Russian Journal of Genetics* **1999,** *35*, 228-232.

*^b^* Cobb, R. E.; Wang, Y.; Zhao, H., High-efficiency multiplex genome editing of *Streptomyces* species using an engineered CRISPR/Cas system. *ACS Synthetic Biology* **2014,** *4* (6), 723-728.
